# Unveiling the abnormal capacity rising mechanism of MoS_2_ anode during long-term cycling for sodium-ion batteries†

**DOI:** 10.1039/d1ra05518f

**Published:** 2021-08-24

**Authors:** Yucheng Zhu, Haoyu Li, Yuanming Wu, Liwen Yang, Yan Sun, Guang Chen, Yang Liu, Zhenguo Wu, Chuhong Zhang, Xiaodong Guo

**Affiliations:** College of Chemical Engineering, Sichuan University Chengdu 610065 Sichuan China xiaodong2009@163.com; School of Mechanical Engineering, Chengdu University Chengdu 610106 Sichuan China; College of Chemistry, Chemical Engineering and Materials Science, Key Laboratory of Molecular and Nano Probes, Ministry of Education, Collaborative Innovation Center of Functionalized Probes for Chemical Imaging in Universities of Shandong, Institutes of Biomedical Sciences, Shandong Normal University Jinan 250014 Shandong China; School of Materials Science and Engineering, Henan Normal University Xinxiang 453007 Henan China; State Key Laboratory of Polymer Materials Engineering, Polymer Research Institute, Sichuan University Chengdu 610065 Sichuan China chuhong.zhang@scu.edu.cn

## Abstract

Transition metal sulfides are considered as one of the most potential anode materials in sodium-ion batteries due to their high capacity, low cost, and rich resources. Among plenty of options, molybdenum sulfide (MoS_2_) has been the focus of research due to the graphene-like layered structure and unique electrochemical properties. Importantly, an abnormal capacity increase phenomenon was observed in the MoS_2_ anode of sodium-ion batteries, but the mechanisms involved are still unclear. In this study, by analyzing the composition and structure of the material after a different number of cycles, we confirmed that the (002) plane shows a significant expansion of the interlayer spacing after the sodium ion insertion process and a phase transformation from the hexagonal phase MoS_2_ (2H-MoS_2_) to the trigonal phase MoS_2_ (1T-MoS_2_). Moreover, the ratio of 1T-MoS_2_ presented an increasing trend during cycling. The dual-phase co-existence leads to enhanced electrical conductivity, higher Na affinity, and higher Na^+^ mobility, thus increasing the capacity. Our work provides a new perspective on the anomalous electrochemical behavior of sulfide anodes during long-term cycling.

## Abstract 2

The capacity rising is due to the biphasic coexistence of MoS_2_ during the cycling and the progressive increase in the 1T-MoS_2_ content. Simultaneously, the layer spacing expanded from 0.62 nm to 1.03 nm during the cycling process.

## Graphical abstract

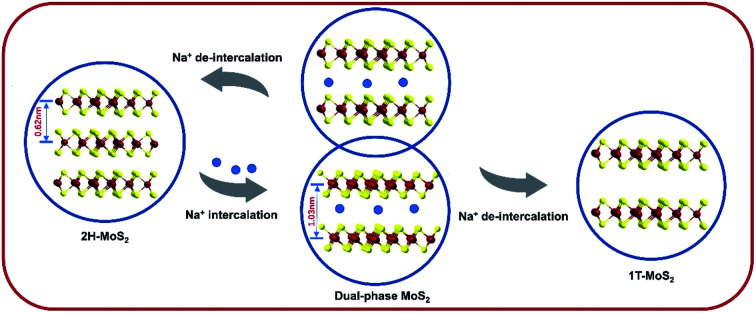

## Introduction

The increase in clean energy has triggered a growing demand for energy storage systems, thus providing an opportunity for the dominance of rechargeable lithium ion batteries (LIBs) owing to their high energy density and long cycle life.^1–4^ However, the intrinsic problems (*e.g.*, rare abundance and uneven geographical distribution of Li) of LIBs make the cost gradually evolve into an obstacle for grid-scale applications.^5–8^ Accordingly, the search for a cost-effective alternative with promising electrochemical performance becomes extremely pressing.^9–11^ Sodium ion batteries (SIBs) with analogical rocking-chair principles to LIBs are emerging as a viable choice for grid-level energy storage and conversion for renewable energy sources due to their low cost, wide global distribution, and excellent electrochemical performance.^12–16^ However, charge storage based on Na^+^/e^−^ is quite challenging to enable both swift and frequent Na^+^ transfer because of the large ionic radius of Na^+^ (1.06 Å *vs.* 0.76 Å for Li^+^).^17–19^ Thus, one of the most urgent issues is exploiting suitable Na^+^-host materials, especially anodic materials, to reach prominent comprehensive electrochemical performances.

Two-dimensional (2D) materials have presented great potential as anode materials in SIBs owing to their fascinating layered structure, which is capable of hosting Na^+^, delivers a large surface area for Na^+^ adsorption, and abundant defects.^13,20–23^ Compared with metal oxides, sulfides, as typical 2D materials, exhibited impressive ability in overcoming such problems (*i.e.*, huge volume variation, severe structure collapse, limited high-rate capability) due to its weakened M–S bonds than M–O bonds.^24^ Among them, MoS_2_ has gained enormous attention because of its fantastic 2D S–Mo–S sandwich-like structure where a single Mo atom is covalently bonded by six S atoms.^25–27^ The laminar MoS_2_ with adjacent planes stacked through the van der Waals forces delivers large interlayer spacing (0.62 nm) for Na^+^ transport.^27^ Conventionally, MoS_2_ in the natural state presented a stable 2H phase with trigonal structure, which endow 2H-MoS_2_ with semi-insulating characteristics with a band gap of ∼1.9 eV, thus delivering a weak conductivity. In contrast, another 1T-MoS_2_ based on tetragonal symmetry with edge-sharing [MoS_6_] octahedra in the metallic phase delivers a much higher conductivity (∼10^7^ times) than 2H-MoS_2_.^28–31^ These merits of MoS_2_ make it an appealing anode material and stimulated much investigation to explore the underlying sodium storage mechanism, especially the distinct rise in capacity during cycling. Some theories were proposed: (1) the decrease in MoS_2_ size along the cycling could boost the infiltration between MoS_2_ and electrolyte.^32^ (2) The electrolyte decomposition could lead to the reversible generation of organic polymeric/gel-like layers and the activation of the MoS_2_ electrode.^33^ (3) The repeated Na^+^ intercalation/deintercalation could result in gradual expanding and interlayer exfoliation.^34^ On the other hand, some investigations revealed that the formation of 1T-MoS_2_ in 2H-MoS_2_ improved the electrochemical performance *via* creating the internal electric field.^33,35,36^ However, so far, there is rarely any systematic study to explore the rise in capacity during cycling in terms of phase transition, which is available to enable a completely different crystalline structure, thus delivering totally distinct sodium storage behavior.

In this work, we synthesized molybdenum sulfide in the form of flowers by hydrothermal synthesis, and annealing at 350 °C allowed the molybdenum sulfide to achieve a stable structure. The structural and compositional changes of the material after long cycling were characterized by X-ray photoelectron spectroscopy (XPS) and transmission electron microscopy (TEM), and it was found that Mo^4+^ peaks showed a significant shift toward lower binding energies after cycling, with a corresponding enhancement of the shift as the number of cycles increased. According to the results of the split-peak fitting and TEM patterns, we confirmed that the MoS_2_ of the metal phase was generated, and its content increased during the cycling process. Also, the layer spacing of some of the molybdenum sulfides widens after the first cycle and remains constant during the subsequent charge/discharge process. These results reveal the influence of structural and compositional changes of molybdenum disulfide on the storage electrodes of long-life sodium ion batteries.

## Experimental section

### Material preparation

Molybdenum sulfide was synthesized by a one-step simple solvothermal method using deionized water as the solvent, ammonium molybdate tetrahydrate as the molybdenum source, and thiourea as the sulfur source. Among them, 1.2 g of ammonium molybdate tetrahydrate ((NH_4_)_6_Mo_7_O_24_·4H_2_O) and 2.2 g of thiourea (CH_4_N_2_S) were dissolved in 35 ml of deionized water and mixed well by stirring for half an hour at room temperature. The solution was then poured into a 100 ml Teflon-lined autoclave, which was maintained at 200 °C for 16 h. After washing with ethanol, it was dried under a vacuum at 80 °C overnight. Finally, it was heated for 4 h at 350 °C under Ar.

### Structural and morphological characterization

The crystalline structure was analyzed from powder X-ray diffraction (XRD, D/Max 2500 PC) with Cu Kα radiation. The XPS spectra were recorded applying an ESCALAB 250Xi (Thermo Fisher) with a monochromatic Al Kα source. Morphologies of the products were investigated by scanning electron microscope (SEM, Zeiss SUPRA 55) and transmission electron microscopy (TEM, JEOL JEM 2100).

### Electrochemical measurements

The working electrode was composed of the active material, acetylene black, and PVDF (Sigma-Aldrich) with a weight ratio of 7 : 2 : 1 dissolved in *N*-methyl-2-pyrrolidinone (Sinopharm). After coating the slurries onto a copper foil current collector and drying at 80 °C overnight, the Cu foil with active material was cut into disk electrodes with a radius of 14 mm. The mass density of the active material in each electrode was 1.0–2.0 mg cm^−2^. The electrochemical measurements of these materials were carried out *via* CR2025 type coin cells assembled in an argon-filled glovebox (Dellis company). The electrolyte was composed of NaCF_3_SO_3_ (1 M) in diglyme and 1 M NaClO_4_ in propylene carbonate (PC) with 5 vol% fluoroethylene carbonate (FEC). Na metal was set as the counter and reference electrodes and glass microfiber as the separator (Whatman, GF/D). The cycling performances of the as-prepared samples were tested on a LAND-BT2013A measurement system with a voltage ranging from 0.4 to 3.0 V at 25 °C.

## Results and discussion

**Fig. 1:**
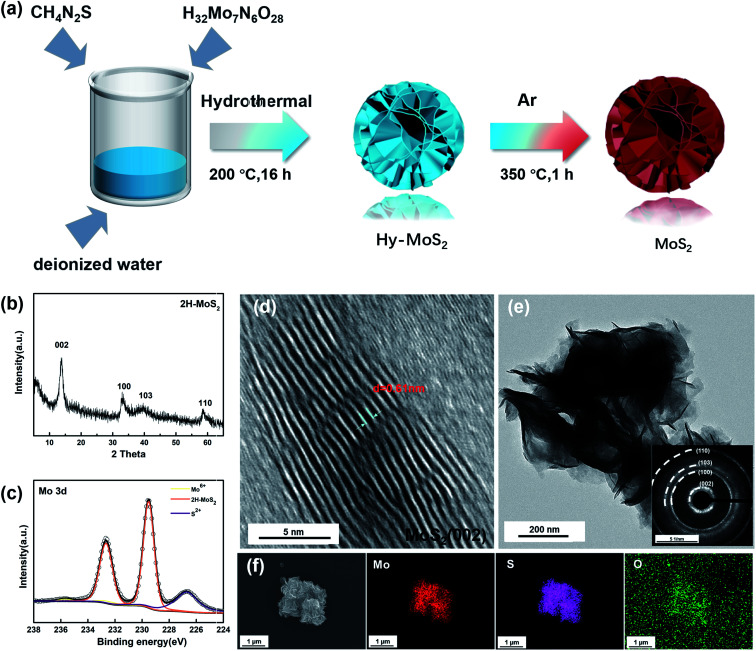
(a) Schematic illustration of flower-like MoS_2_ through a hydrothermal technique. (b) XRD patterns of 2H-MoS_2_. (c) High-resolution XPS of Mo 3d. (d) HRTEM images of the nanostructured MoS_2_. (e) SAED pattern of MoS_2_. (f) SEM images of MoS_2_ and mapping images (Mo, S, and O) of MoS_2_.

The crystal structures of as-prepared MoS_2_ were examined by X-ray diffraction (XRD). Fig. 1b displays four major diffraction peaks at 13.6, 33.2, 39.7, and 58.7° assigned to the (002), (100), (103), and (110) planes of 2H-MoS_2_ (JCPDS No. 37-1492). Based on the main diffraction peak at 13.6°, the interlayer distance of MoS_2_ is 0.61 nm *via* calculation by Bragg's Law. According to the previous reports, XPS can be used to identify the 1T phase in the sample because Mo 3d spectra of the 1T phase will shift towards lower binding energy. As shown in Fig. 1c, the two peaks of the annealed sample are located at 229.7 eV and 232.6 eV, there is no offset of the peak, and the results indicate that the annealed sample is pure 2H-MoS_2_. Inevitably, the two faint peaks show the presence of Mo^6+^ and S 2p, which are located at 235.7 eV and 226.7 eV, indicating that inevitable contact with air during the synthesis and preservation process caused partial oxidation of MoS_2_.^37^

The morphology and structure of the samples were observed by SEM, TEM, and high-resolution TEM. In Fig. 1d, we can observe that the layer spacing of the (002) plane is roughly 0.61 nm, which is consistent with the position of the (002) diffraction peak in XRD. The low magnification TEM (Fig. 1e) shows the different layers in the sample. The thinner edge areas are more susceptible to oxidation, and it can be judged that the particle size is about 500 nm. The (002), (100), (103), and (110) planes in the selected area electron diffraction pattern (Fig. 1e) also correspond to the four peaks in the XRD patterns. As shown in Fig. 1f, the sample has a flower-like structure, and S and Mo are uniformly distributed throughout the particles. Moreover, a clear distribution of O on the particles can also be observed, which also confirms that the samples were indeed partially oxidized.

**Fig. 2:**
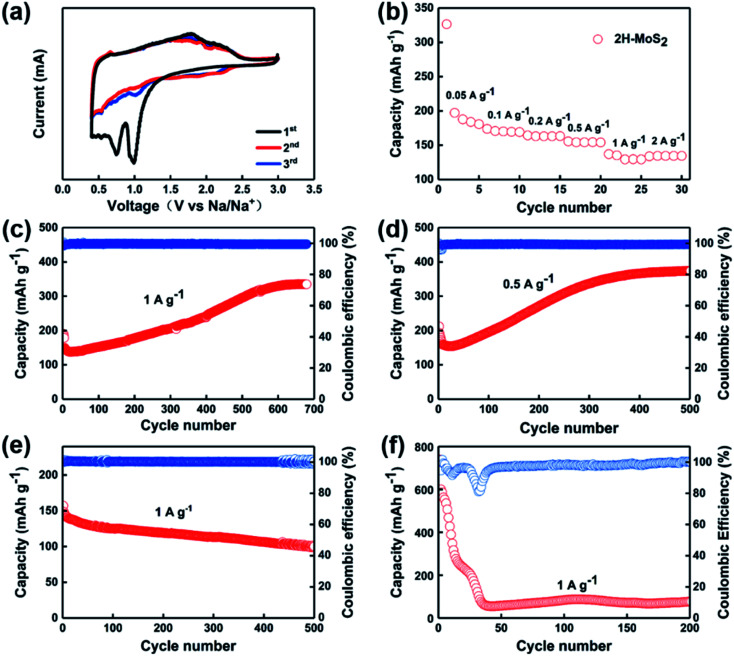
(a) Cyclic voltammetry curves of the as-prepared MoS_2_. (b) Rate capability of the as-prepared MoS_2_. (c) and (d) Cycling performances of as-prepared MoS_2_ nanosheets at 1 A g^−1^ and 0.5 A g^−1^. (e) Cycling performances of as-prepared MoS_2_ nanosheets at 1 A g^−1^ with 1 M NaClO_4_ in propylene carbonate (PC) with 5 vol% fluoroethylene carbonate. (f) Cycling performance of as-prepared MoS_2_ nanosheets at 1 A g^−1^ with a cut-off voltage in the range of 3–0.01 V.

According to the previously reported studies, the cut-off voltage affects the charge/discharge performance of the battery because conversion reactions occur below 0.4 V, which leads to poor reversibility of the whole process.^34,38^ Intercalation and conversion reaction are shown in eqn (1) and (2), which are similar to that of LIBs.^39,40^ Therefore, during electrochemical tests, we have chosen the voltage range of 3.0–0.4 V for our experiments.

1 MoS_2_ + *x*Na = Na_*x*_MoS_*x*_ (above 0.4 V, *x* < 2)

2 Na_*x*_MoS_*x*_ + (4 − *x*)Na = Mo + 2Na_2_S (below 0.4 V)

In order to study the redox reactions occurring during the charge and discharge process, CV tests were performed on the samples. As shown in Fig. 2a, there are two cathodic peaks at 0.92 V and 0.76 V, respectively, during the first discharge, which corresponds to the process of Na^+^ inserting into MoS_2_ and accompanied by the occurrence of phase transition processes. The peaks at positions 1.9 V and 2.2 V appearing during charging indicate the process of Na^+^ extracting from Na_*x*_MoS_2_.^31,38^ The excellent overlap shown in the curves of both the second cycle and the third cycle confirms the ideal reversibility of the sample. Fig. 2b shows the rate performances of the electrodes. The reversible capacity of the MoS_2_-based anode cell can be obtained as 175, 170, 164, 154, 135, and 134 mA h g^−1^ at the current densities of 0.05, 0.1, 0.2, 0.5, 1, and 2 A g^−1^, respectively. Fig. 2c and d show the cycling stability of MoS_2_ as anode for SIBs at a current density of 1 A g^−1^ and 0.5 A g^−1^, respectively. Interestingly, after continuous reduction of capacity after the first 30 cycles, the capacity of the MoS_2_ anode showed an anomalous rise. The first discharge mass specific capacity of MoS_2_ at 1 A g^−1^ is 198 mA h g^−1^. After 30 cycles, the capacity drops to nearly 150 mA h g^−1^, which can be attributed to the generation of the SEI and the activation process of the batteries.^41^ However, the capacity of the MoS_2_ anode completely reversed the previous decreasing trend after the phenomena taking place at the 30th cycle. The capacity rose in a steady manner until it stabilized after 650 cycles, as it rose from 134 mA h g^−1^ to around 340 mA h g^−1^. In the subsequent cycles, the capacity stays stable and remains at 340 mA h g^−1^ with the generation of Na_1.0_MoS_2_.^42^ Similarly, the same situation of capacity falling and then rising occurs at a current of 0.5 A g^−1^. The discharge capacity is 215 mA h g^−1^ for the first cycle and then decreases to 150 mA h g^−1^ after 20 cycles. During the subsequent cycles, the capacity rises to 370 mA h g^−1^ after about 400 cycles and remains stable. On the contrary, there was no abnormal rise in capacity when 1 M NaClO_4_ in propylene carbonate (PC) with 5 vol% fluoroethylene carbonate was applied as the electrolyte (Fig. 2e). The ether electrolyte itself inhibits the shuttle effect of the generated polysulfide, and the use of ether electrolyte instead of ester electrolyte can reduce the organic components contained in the negative SEI film of the sodium ion battery so that the interface formed between the SEI film and the negative material is more dense and stable.^43,44^ Meanwhile, when the cut-off voltage was in the range of 0.01–3 V, the capacity dropped steadily from 739 mA h g^−1^ to 76 mA h g^−1^ due to an irreversible conversion reaction ranging from 0.4 V to 0.1 V (Fig. 2f). The reason for the phenomenon is mainly the formation of Mo and S monomers, which cannot completely regenerate MoS_2_ during cycling.

The anomaly of capacity rising is a relatively common phenomenon in MoS_2_ electrodes, and a number of explanations have been given based on different aspects. Along with the increase in capacity, a decrease in impedance is also observed. It is interesting to note that this phenomenon is usually limited to the use of ether electrolytes with controlled cut-off voltage intervals.

**Fig. 3:**
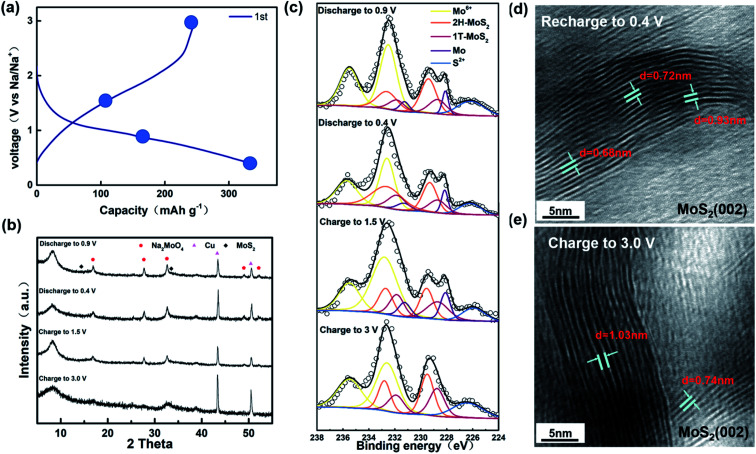
(a) Charge/discharge curves of the MoS_2_ electrode for the first cycle at a current density of 0.02 A g^−1^. (b) and (c) *ex situ* XRD and XPS pattern of different charge/discharge depths. (d) and (e) HRTEM of MoS_2_ after recharging to 0.4 V and charge to 3.0 V.

As shown in Fig. 3a, we chose four points to test during the first cycle of charging and discharging at a current of 0.02 A g^−1^. Combined with the position of the peak in the first-turn CV, we chose four points for characterization tests for discharging to 0.9 V, 0.4 V and charging to 1.5 V, 3.0 V. In the *ex situ* XRD results (Fig. 3b), we can see that the position of peak (002) has shifted about 9° left from 14°. The offset is universally considered to be caused by the expansion of the layer spacing of the (002) plane.^45,46^ In previous reports, the phenomenon and corresponding explanation that the layer spacing of MoS_2_ expands due to the insertion of Na^+^ were observed and concluded by researchers through some characterizations.^47^ Surprisingly, the expanded layer spacing after going through the Na^+^ insertion process was not reduced by Na^+^ extraction. The graph shows that the (002) peak position also remains stable at around 9° when charged to 1.5 V and 3.0 V. Combined with Fig. 3d and e, it can be confirmed that the layer spacing is not restored during the charging process after the expansion. Furthermore, the *ex situ* XRD diagram shows the presence of Na_2_MoO_4_ due to partial MoS_2_ oxidation as well. According to Michael *et al.*, MoS_2_ is more likely to be present in the form of MoO_4_^2+^ rather than MoO_3_ when oxidized.^48^ In the *ex situ* XPS, we can clearly see a peak of Mo at 235.5 eV, again proving that some MoS_2_ was oxidized during the cycle. Curiously, in the *ex situ* XPS for the samples discharging to 0.9 V and 0.5 V and charging to 1.5 V, we can see a clear peak at 228.0 eV, showing the existence of Mo, which is not consistent with the previous paper. We speculate that it is because some of the molybdenum oxide produced Mo during the reaction. It is confirmed that no chemical reaction occurred after assembling the battery without discharging and just leaving it for 12 h (Fig. S1†). After one cycle, the XPS of the sample charged to 3.0 V shows that the material consists of Mo^4+^ and Mo^6+^, but the Mo^4+^ peak is shifted toward the lower binding energy with respect to the original sample. Combined with what is shown in Fig. S2† and the previous reports, it is confirmed that there is 1T-MoS_2_ generation.^49–51^ Through heating or aging, unstable 1T-MoS_2_ will usually undergo phase transition back to 2H-MoS_2_.^52,53^ However, from the *ex situ* XPS analysis, it is obvious that the 1T-MoS_2_ produced by the phase transition does not return to the 2H-MoS_2_ phase but is basically stable in the sample. From the composition, the material after one cycle charge and discharge process contains Na_2_MoO_4_, 1T-MoS_2_, and 2H-MoS_2_. According to Fig. S3,† we can clearly see that the material composition after 200 cycles remains the same as that after the first charge and discharge process. The intercalation of Na^+^ tends to enhance the stability of 1T-MoS_2_, as shown in Fig. 3c. It can be clearly observed that there is still a co-existence of two phases, and the experimental results show that 1T-MoS_2_ still is stable and exists after Na^+^ is extracted. The explanation, which Xu proposed that MoO_3_ can also stabilize 1T-MoS_2_ without phase change, also applies to our experimental results.^54^ It is consistent with the calculation of Wang *et al.* that 1T-MoS_2_ is the dominant phase after Na^+^ extraction.^55^

**Fig. 4:**
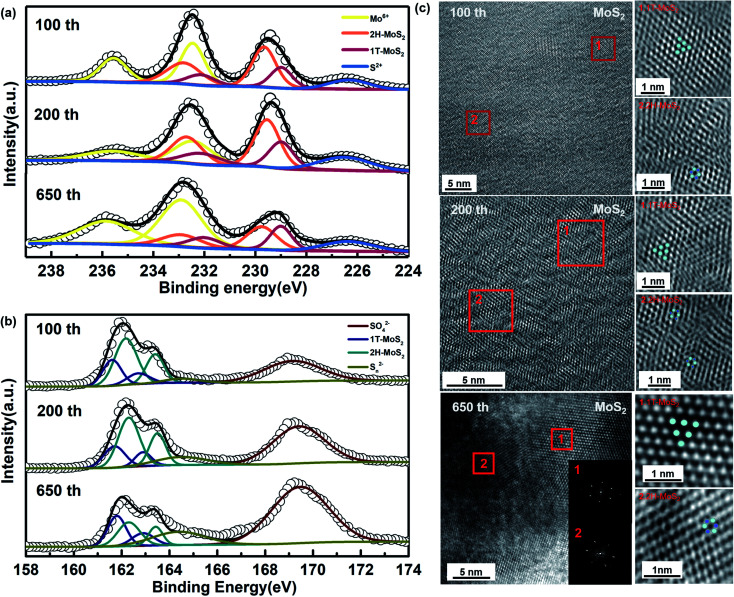
(a) and (b) *Ex situ* XPS of MoS_2_ anode after charge/discharge for different cycles, (c) HRTEM patterns and the corresponding SAED pattern of MoS_2_ anode after 650 cycles.

In a subsequent study, we have tried to analyze the composition of phases in a different number of cycles. According to Fig. 2c and d, the capacity rising continues until the cycling reaches stability at around 600 cycles. The cycling process of the first lap has been analyzed, and the partial phase change during its recharge/discharge process has been confirmed. We selected cells after cycling 100th and 200th during the capacity rising to discover their composition and morphology. As shown in Fig. 4a and b, it is clearly observed that there is still a co-existence of the two phases. Furthermore, as shown in the diagram, there is always the presence of Mo^6+^, and combined with Fig. S3† we can determine its component as MoO_4_^2−^. The Na_2_MoO_4_ in the cathode material provides good protection to the electrode and contributes to better cycling stability.^54,56^ According to Fig. 4c, the same two phases co-exist after 100 cycles, 200 cycles, and 650 cycles, in agreement with the results of *ex situ* XPS. It can be seen that the 1T (octahedral) and 2H (trigonal) phases co-exist in the same nanosheet, which can be classified as type IV with a 2D in-plane 1T/2H MoS_2_ heterogeneous structure.^20,57^ There are no significant defects in the boundaries between them. Meanwhile, the petal-like structure can still be observed in the morphology of the whole material (Fig. S4†).

According to Fig. S5,† there is a significant widening of the layer spacing on the (002) surface, and this widening appears and is maintained from the first cycle onwards. From this aspect, there is a relationship between the increase in capacity and the expansion of the layer spacing. Meanwhile, when analyzed in terms of composition, it can be found that the Mo^4+^ peaks show a significant shift toward lower binding energies, which means that the ratio of MoS_2_ in different phases has changed. From the 1st cycle to the 650th cycle, the 1T phase appears and continues to increase, showing the same trend as the rising capacity. It is noteworthy that S 2p_3/2_ and S 2p_1/2_ show the same peak shift in agreement with Mo^4+^ (Fig. 4b). Moreover, the generation of metallic phases as well as the increase in content lead to a continuous increase in capacity.

According to the results, the appearance and increase in the amount of 1T-MoS_2_ can be determined. The contribution of 1T-MoS_2_ to the capacity is usually explained in terms of these aspects. Benefiting from different coordination between Mo and S atoms, the 1T-MoS_2_ has a much higher electronic conductivity than the semiconducting 2H counterpart. Moreover, 1T-MoS_2_ and 2H-MoS_2_ could induce an internal electric field at the corresponding heterointerface because of the different Fermi energies. The phase change reduction is also an important reason for the capacity increase. During the discharge, a phase transition process from 2H-MoS_2_ to 1T-MoS_2_ occurs, but after the partial 1T phase stabilization, only the embedding process of sodium ions and no more phase transition occurs. According to the reports, the process is particularly similar to SnS, which has superior cycling performance relative to SnS_2_ during cycling because it undergoes only two phase transitions less than the three phase transformation of SnS_2_.^58^

## Conclusions

In general, we synthesized pure 2H-MoS_2_ by hydrothermal method and applied it as the anode material for sodium-ion batteries. We controlled the charge/discharge cut-off voltage and used the DME electrolyte, and the battery capacity showed an abnormal increase. Based on the analysis of electrode sheets after cycling for a different number of revolutions, we found that 1T-MoS_2_ was generated after cycling and its content tended to increase with capacity during cycling. By analyzing the effect of 1T-MoS_2_, the capacity rising is due to the formation of a biphasic co-existence of MoS_2_ during the cycle and the progressive increase in the 1T-MoS_2_ content. At the same time, the layer spacing expanded from 0.62 nm to nearly 1.03 nm during the cycling process, which also contributed to the capacity of the cell. Finally, this study provides an explanation for the anomalous phenomenon of transition metal sulfides as anode for SIBs, which provides a new method and idea for future research on transition metal sulfides.

## Conflicts of interest

There are no conflicts to declare.

## Supplementary Material

RA-011-D1RA05518F-s001
