# Supplementary material for: Unveiling the abnormal capacity rising mechanism of MoS2 anode during long-term cycling for sodium-ion batteries
Source: RSC Adv. 2021 Aug 24;11(46):28488–95. doi: 10.1039/d1ra05518f (PMC9038034; doi:10.1039/d1ra05518f)
Supplement: RA-011-D1RA05518F-s001 [file RA-011-D1RA05518F-s001.pdf]

Electronic Supplementary Material (ESI) for RSC Advances.
This journal is © The Royal Society of Chemistry 2021

Unveiling the Abnormal Capacity-Rising Mechanism of MoS_2 Anode during Long-Term Cycling for Sodium-Ion Batteries

Yucheng Zhu[a], Haoyu Li[a], Yuanming Wu[a], Liwen Yang[a], Zuguang Yang[a], Yan Sun[b], Guang Chen[c], Yang Liu[d], Zhenguo Wu[a], Chuhong Zhang[e], Xiaodong Guo[a],

[a] College of Chemical Engineering, Sichuan University, Chengdu 610065, Sichuan, China

[b] School of Mechanical Engineering, Chengdu University, Chengdu 610106, Sichuan, China

[c] College of Chemistry, Chemical Engineering and Materials Science, Key Laboratory of Molecular and Nano Probes, Ministry of Education, Collaborative Innovation Center of Functionalized Probes for Chemical Imaging in Universities of Shandong, Institutes of Biomedical Sciences, Shandong Normal University, Jinan 250014, Shandong, China

[d] School of Materials Science and Engineering, Henan Normal University, Xinxiang 453007, Henan, China

[e] State Key Laboratory of Polymer Materials Engineering, Polymer Research Institute, Sichuan University, Chengdu 610065, Sichuan, China

*Corresponding authors.
E-mail addresses: xiaodong2009@163.com (Xiaodong Guo);
chuhong.zhang@scu.edu.cn (Chuhong Zhang).

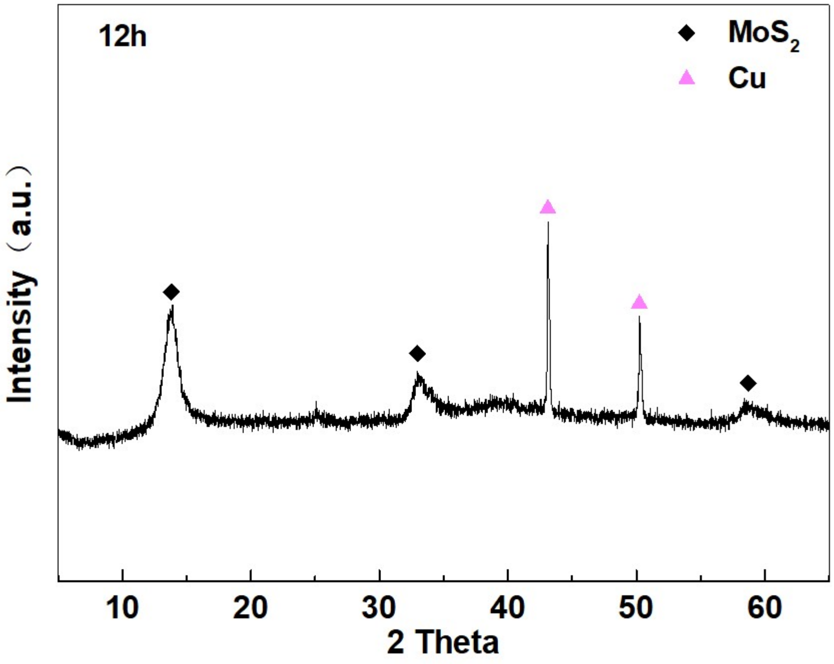

Fig. S1 XRD patterns of 2H-MoS_2 resting for 12h under battery environment.

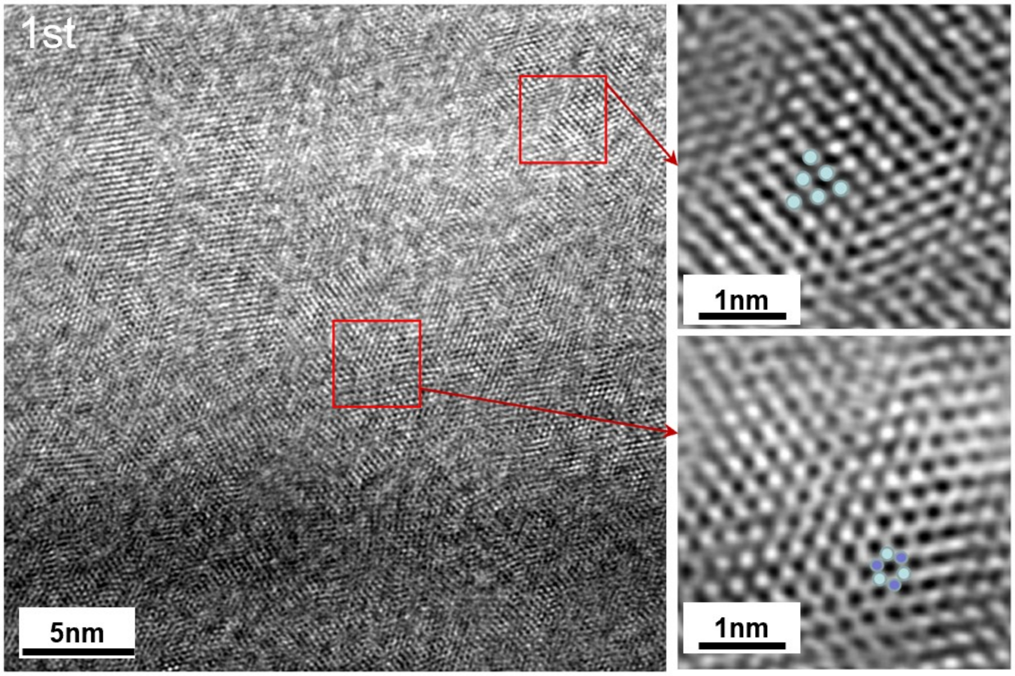

Fig. S2 HRTEM patterns pattern of MoS_2 after first charge/discharge process

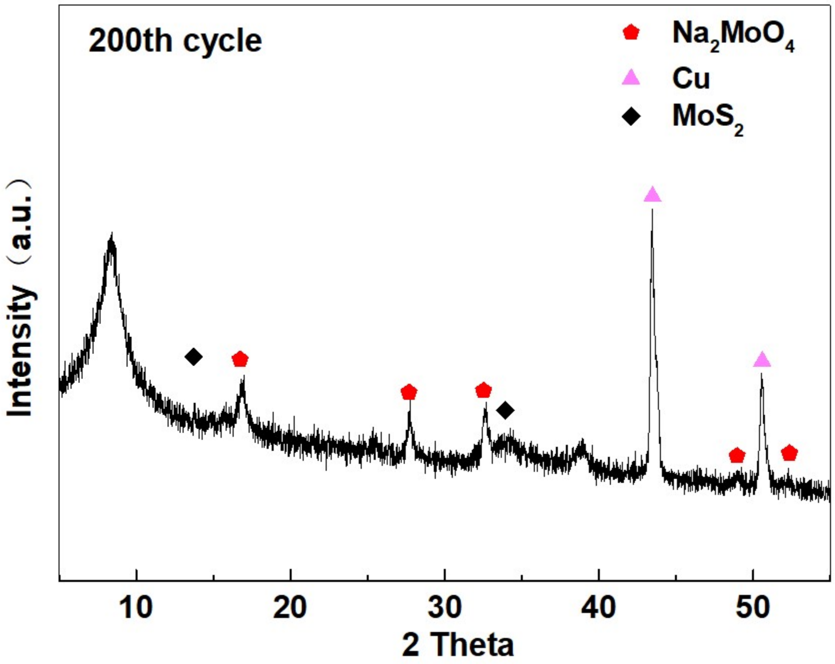

Fig. S3 XRD patterns of 2H-MoS2 after 200th cycle

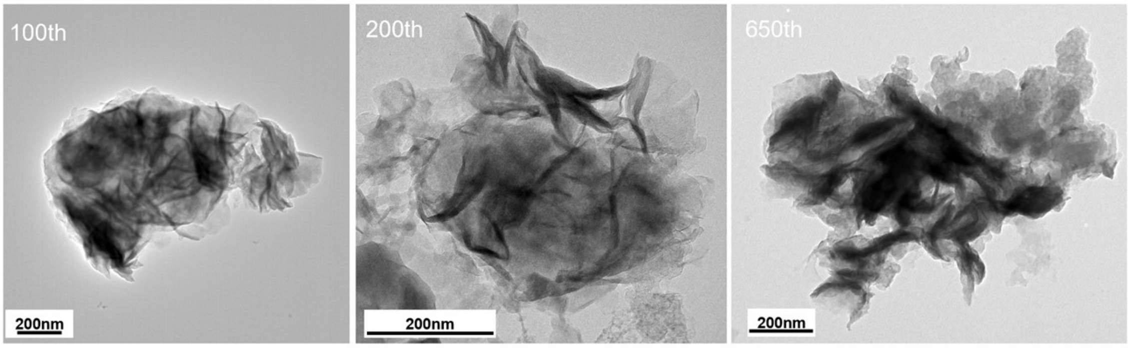

Fig. S4 HRTEM patterns of MoS_2 anode after100 cycles, 200 cycles and 650 cycles

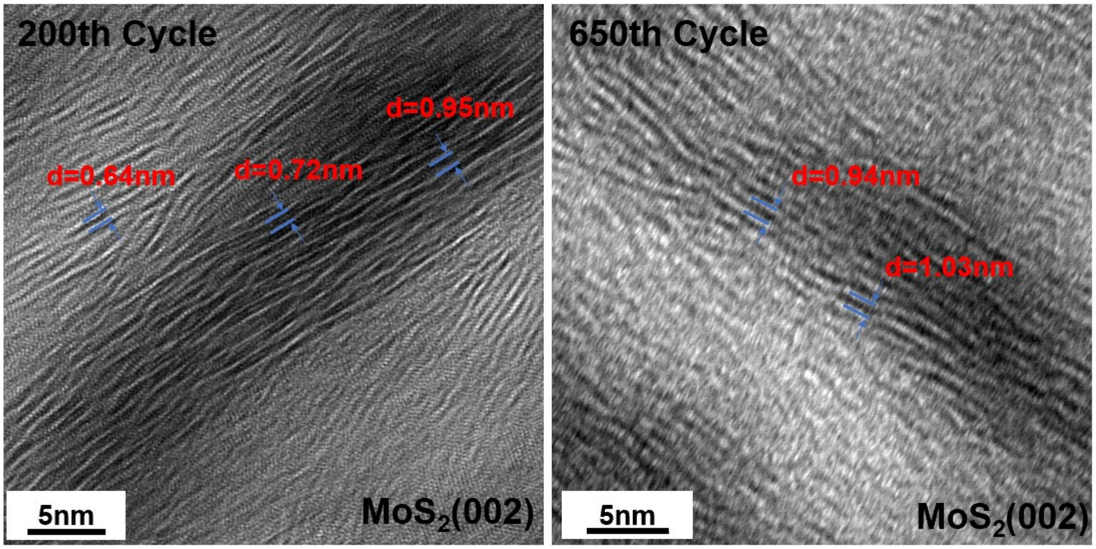

Fig. S5 HRTEM patterns of MoS_2 anode after 200th cycle and 650th cycle
